# Microarray study of gene expression profile to identify new candidate genes involved in the molecular mechanism of leptin-induced knee joint osteoarthritis in rat

**DOI:** 10.1186/s41065-017-0039-z

**Published:** 2017-07-04

**Authors:** Qing Fan, Zhu Liu, Chao Shen, Hai Li, Jing Ding, Fangchun Jin, Lin Sha, Ziming Zhang

**Affiliations:** 1 Departments of Pediatric Orthopedics, Xin Hua Hospital Affiliated to Shanghai Jiaotong University, School of Medicine, 1665 Kongjiang Road, Yangpu, Shanghai, 200092 China; 2 Departments of Orthopedics, Xin Hua Hospital Affiliated to Shanghai Jiaotong University, School of Medicine, Shanghai, 200092 People’s Republic of China

**Keywords:** Osteoarthritis, Leptin, Rats, DEG

## Abstract

**Background:**

Osteoarthritis (OA) is one of the most prevalent chronic joint diseases while the precise genetic mechanism remains elusive. In this study, we investigated the gene expression profile in OA by microarray analysis.

**Results:**

Histopathological characteristics of OA cartilage were examined using a rat model of leptin-induced OA. Gene expression profile of leptin-induced articular cartilage and healthy rat cartilage were compared using genome-wide microarray hybridization. A total of 1857 genes differentially expressed genes (1197 upregulated and 660 downregulated) were identified, some of which are known to be associated with leptin-induced OA phenotype. These included genes related to MMPs, inflammatory factors, growth factors, apoptotic genes and osteogenic genes. In addition, upregulated expressions of some new candidate genes, which have hitherto fore not been linked to OA (such as *BCL2L11*) were detected in leptin-induced OA cartilage, which suggests that these genes might be important for OA molecular mechanism.

**Conclusion:**

Our findings suggest that pathogenesis of leptin-induced OA involves modulation of expression of multiple genes, although the underlying molecular mechanisms need to be studied further. Further investigation of leptin-induced gene expression changes is needed to gain new insights into the molecular mechanism of OA pathogenesis.

**Electronic supplementary material:**

The online version of this article (doi:10.1186/s41065-017-0039-z) contains supplementary material, which is available to authorized users.

## Background

Osteoarthritis (OA) is one of the most prevalent musculoskeletal disorders and is the most common form of arthritis among the elderly population [1]. Multiple factors are involved in the onset and progression of OA. Among the OA phenotypes associated with joint failure, such as malalignment, muscle dysfunction, ligament tear, subchondral bone sclerosis and osteophyte growth, cartilage degradation is the most common phenotype [2]. Chondrocytes, which is the only cell type in healthy cartilage tissue, are critical for remodeling and maintenance of the structure and function of the cartilaginous extracellular matrix. Over the last few years, chondrocyte apoptosis and the related signaling pathways have evoked much interest, since it is considered to be the leading mechanism of cartilage degradation in OA joint [3–5]. Although the underlying mechanisms involved in pathological progression has remained elusive, inhibition of chondrocyte death has been suggested as a therapeutic target for limiting cartilage degradation. Furthermore, inflammation is believed to play a critical role in the onset of OA due to its catabolic effect on cartilage [6]. Systemic and local production of inflammatory cytokines, activation of complement and innate immune response are shown to be important in the progression of OA.

Obesity has been traditionally considered as a local risk factor for OA due to increased mechanical load on weight-bearing joints such as knees and hips [7]. But the fact that obese individuals are also at a higher risk of OA in non-weight-bearing joints such as hands, wrists and shoulders [8] suggests that additional factors related to obesity may also play a role in the onset and progression of OA. It has been reported that leptin, which is produced predominantly in white adipose tissue, shows significantly higher levels in synovial fluid of patients with OA as compared to that in healthy individuals. This leads us to an epidemiological link between OA and leptin [9, 10]. Since functional leptin receptor is found on healthy chondrocytes [11], it is believed that leptin/leptin receptor signaling axis may modulate synovial joint homeostasis. During OA progression, excessive concentration of leptin may induce metabolic changes in chondrocytes. Moreover, leptin has also been reported to modulate inflammatory factors and catabolic enzymes in cartilage and other joint tissues [12]. Simopoulou et al. reported that leptin inhibited chondrocyte proliferation and increased IL-1β, MMP-9 and MMP-13 secretion to the synovial fluid. Insights gained from these studies indicate a temporal relationship of leptin with onset as well as progression of OA. Since leptin is a catabolic factor in cartilage metabolism and could be a driving factor in the pathogenesis of OA, intra-articular injection of leptin has been used to establish a rat model of OA [13].

High through-put gene expression studies such as microarray analyses are increasingly used to identify changes in gene expression profiles during the onset and progression of complex diseases such as OA. Further, these have also been used to identify biomarkers to aid diagnosis as well as to monitor disease activity and therapeutic response [14, 15]. Several microarray studies have recently been performed to study OA related gene expression, with a focus on cartilage of OA patients [16–20]. These findings can help understand the pathogenetic mechanisms involved in progression of arthritis. However, innate differences between patients, such as with respect to age, and stratification of joints, tend to introduce an element of bias. Therefore, assessment of gene expression changes in response to external stimuli, such as leptin, may provide new insights into the genetic determinants of OA and the signaling pathways involved in its pathogenesis.

In this study, we integrated microarray data from articular chondrocytes of rats and sought to identify gene expression profiles and signaling pathways that can mark the pathogenetic changes in OA cartilage. To the best of our knowledge, this is the first study to perform microarray analyses to compare healthy cartilage and leptin-stimulated cartilage, thereby providing clues to the pathogenetic mechanisms of OA. The results of this study could help identify new diagnostic markers and therapeutic targets.

## Methods

### Experimental animals

Male Sprague–Dawley (160–180 g) rats (age, 6 weeks) were purchased from the Experimental Animal Center at the Shanghai Jiao Tong University (Shanghai, China). All experiments involving use of live animals were approved by the University Animal Care and Use Committee. Animals had ad libitum access to water and food. Before the experiment, all rats were acclimatized for 6 days in the facility.

Rats were randomly divided into two groups: control (*n* = 12) and leptin-induced (*n* = 12). Before injection, all rats were anesthetized with chloral hydrate. Rats in the control group were administered 50 μL sterile saline solution into the right knee joint. Rats in the leptin-induced group were administered rat recombinant leptin (100 μg) (R&D Systems, Minneapolis, MN) into the right knee joint. Forty eight hours after injection, six rats from each group were euthanized for RNA extraction. The cartilage of knee joint was collected, snap-frozen in liquid nitrogen [21], and stored at −80 °C in an ultra-low temperature freezer until further use. The remaining six rats were kept alive until 4 or 8 weeks post-injection for histological examination.

### Histological examination

Three rats from each group were euthanized at 4 weeks post- injection while the last 3 rats from each group were euthanized at 8 weeks post-injection. Right knee joints of rats were harvested. After fixation in 10% neutral buffered formalin for 48 h, all samples were immerged in hydrochloric acid solution for 24 h for decalcification. The decalcified tissues were subjected to sequential dehydration in a graded alcohol series, paraffin-embedded, and 5-μm sections prepared with a microtome. The sections were stained with Safranin O and independently examined by two pathologists. OARSI score was obtained to evaluate the severity of osteoarthritis [22].

### RNA extraction

Cartilage from the two groups of rats was harvested in RNase-free conditions immediately after euthanasia, snap-frozen in liquid nitrogen and stored at −80 °C in an ultra-low temperature freezer. Total RNA was extracted with use of Trizol (Invitrogen, Carlsbad, CA) and purified using RNeasy RNA isolation kit (Qiagen, Germany). The RNA concentration was measured by a Nanodrop spectrophotometer (ThermoFisher, USA). RNA integrity was assessed with Agilent 2100 Bio-analyzer (Agilent Technologies, Palo Alto, CA).

### Microarray hybridization and data analysis

Microarray hybridization was performed as described elsewhere [23]. Briefly, the Cy3-labeled cRNA with whole rat Genome oligo Microarray (Agilent Technologies, Santa Clara, CA) was hybridized using the Gene Expression Hybridization Kit (Agilent Technologies, Santa Clara, CA) in a hybridization oven (Agilent Technologies). Raw data were obtained and normalized by the Quantile algorithm, Gene Spring Software 11.0 (Agilent Technologies, Minnetonka, MN). After normalization, gene-expression changes in the leptin-induced group which showed at least a 2.0-fold change from that in the control group were defined as up-regulated or down-regulated. To determine significant proportions of differentially expressed genes within treated groups, the hypergeometric probability *p* was calculated. *P* < 0.05 was considered statistically significant.

### Analysis of differentially expressed genes, gene ontology and cell signaling pathways

We used a linear model for microarray data (LIMMA) and the empirical Bayes method to identify and compare the DEGs in the control and leptin-induced groups. DEGs were defined as significant if both the statistical *p* value was *P* < 0.05 and the fold change (FC) were >2.0. Gene ontology (GO) annotations were downloaded from internet (http://www.geneontology.org and http://www.ncbi.nlm.nih.gov). All GO categories with *p* value < 0.05 were listed. The database of signaling pathway analysis was also obtained from the internet (http://www.genome.jp/kegg); pathway categories with *p* < 0.05 are reported.

### Quantitative reverse transcriptase PCR analysis

The microarray data was verified by reverse transcriptase quantitative PCR (RT-qPCR). Ten representative genes were selected from the list of differentially expressed genes. Total mRNA was extracted from 6 different rats. The RNA was reverse transcribed to synthesize cDNA using the RevertAid™ cDNA Kit (ThermoFisher, San Diego, CA). Then, PCR was carried out with the SYBR grenn Real Master mix (Tiangen, Beijing, China) and a LightCycler (Roche Diagnostics, Germany). Priming specificity was validated by testing melting curve of the PCR products. β-actin was used as reference gene for analysis. The relative expression level was calculated with the 2 ^–ΔΔCt^ method.

### Statistical analysis

All quantitative data are presented as mean ± standard error. Between-group differences were assessed with Student *t*-test. Multi-group (≥3) comparisons were performed with one-way Analysis of Variance (ANOVA) followed by Scheffe’s test. *p < 0.05* was considered to be statistically significant.

## Results

### Leptin induces inflammation and degradation of articular cartilage

**Fig. 1:**
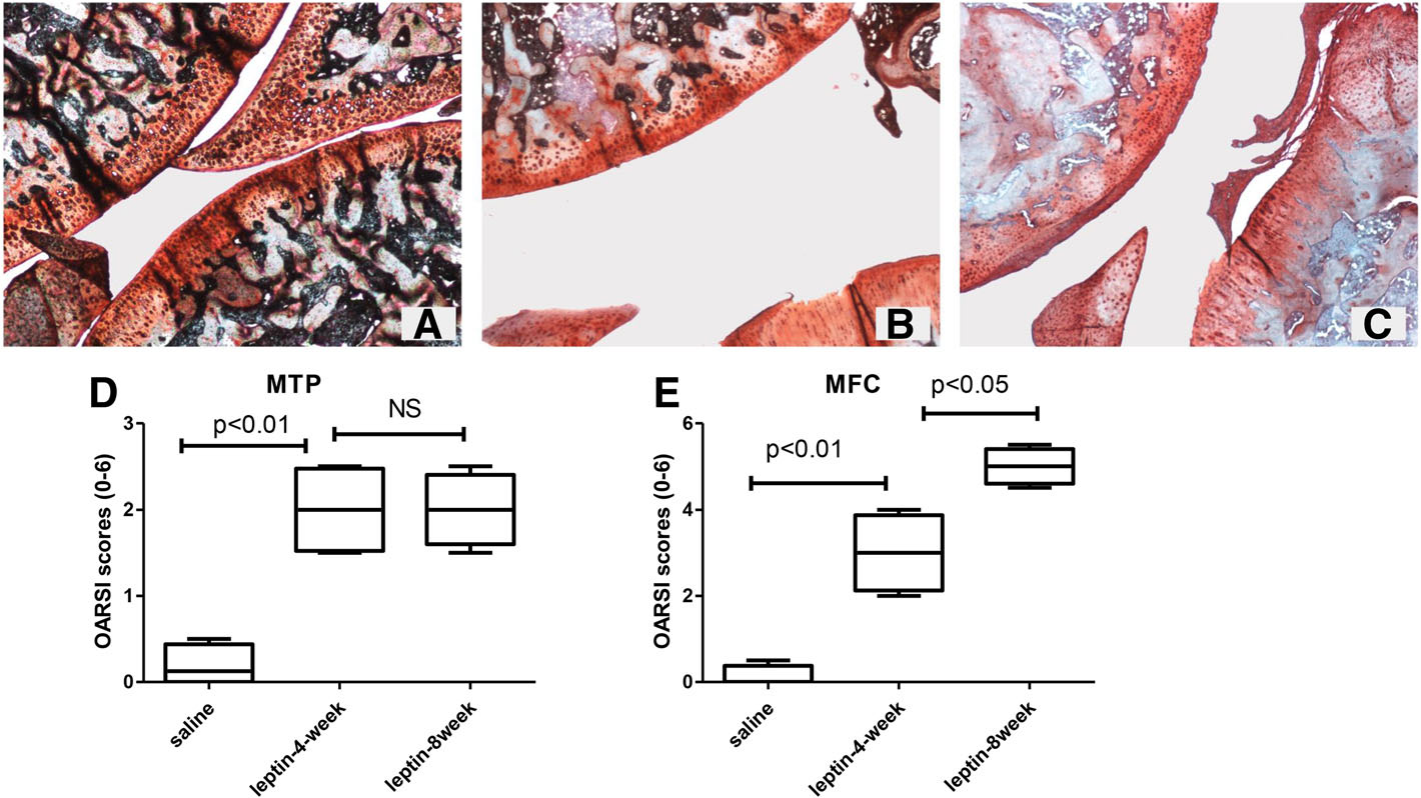
Histological examination of leptin-induced cartilage and healthy cartilage. Safranin O−/fast green−/hematoxylin-stained midsagittal sections of knee joints of rats in the control group (**a**), leptin-induced group at 4 weeks (**b**) and leptin-induced group at 8 weeks (**c**). Articular cartilage tissue in leptin-induced group lost glycosaminoglycans not only in the superficial zone but also down deep in the middle zone. Original magnification × 40. (**d**-**e**) OARSI score was obtained to quantify the severity of cartilage degradation. Data are presented as a *box* and *whisker diagram*. *Upper* and *lower bars* represent maximum and minimum. Edges of the box represent the *first quartile*, *median* and *third quartile*. The *line* in the middle represents the median. NS = Not significant; MTP = medial tibial plateau; MFC = medial femoral condyle. *P* values calculated by one-way analysis of variance followed by Scheffe’s test

Safranin O staining marks proteoglycan/glycosaminoglycans (GAG) of hyaline cartilage, and is widely used to detect proteoglycan/GAG content in articular cartilage [24]. In this study, histological examination (Safranin O staining) of cartilage on tibial plateaus in rat was performed in both leptin-injection and control groups. In the control group, the superficial zone of the cartilage was smooth and intact. Positive staining of Safranin O indicated no loss of proteoglycan in the mid- or deep zones (Fig. 1a). In contrast, cartilage from leptin-injection group showed proteoglycan depletion from the superficial zone to the mid-zone, 4 weeks after intra-articular injection of leptin (Fig. 1b). Furthermore, cartilage from leptin injection group displayed early-stage osteoarthritic phenotype, including swelling, superficial destruction and extensive loss of proteoglycan staining at 8 weeks post -injection (Fig. 1c). We also determined OARSI score as a measure of the severity of osteoarthritis. Scores for both medial tibial plateau and medial femoral condyle in the leptin-induced group were significantly higher than those in the control group. These results suggest that leptin may have induced the onset of OA by reducing the GAG content in the cartilage matrix.

### Differential gene expression between health and leptin-induced cartilage

**Fig. 2:**
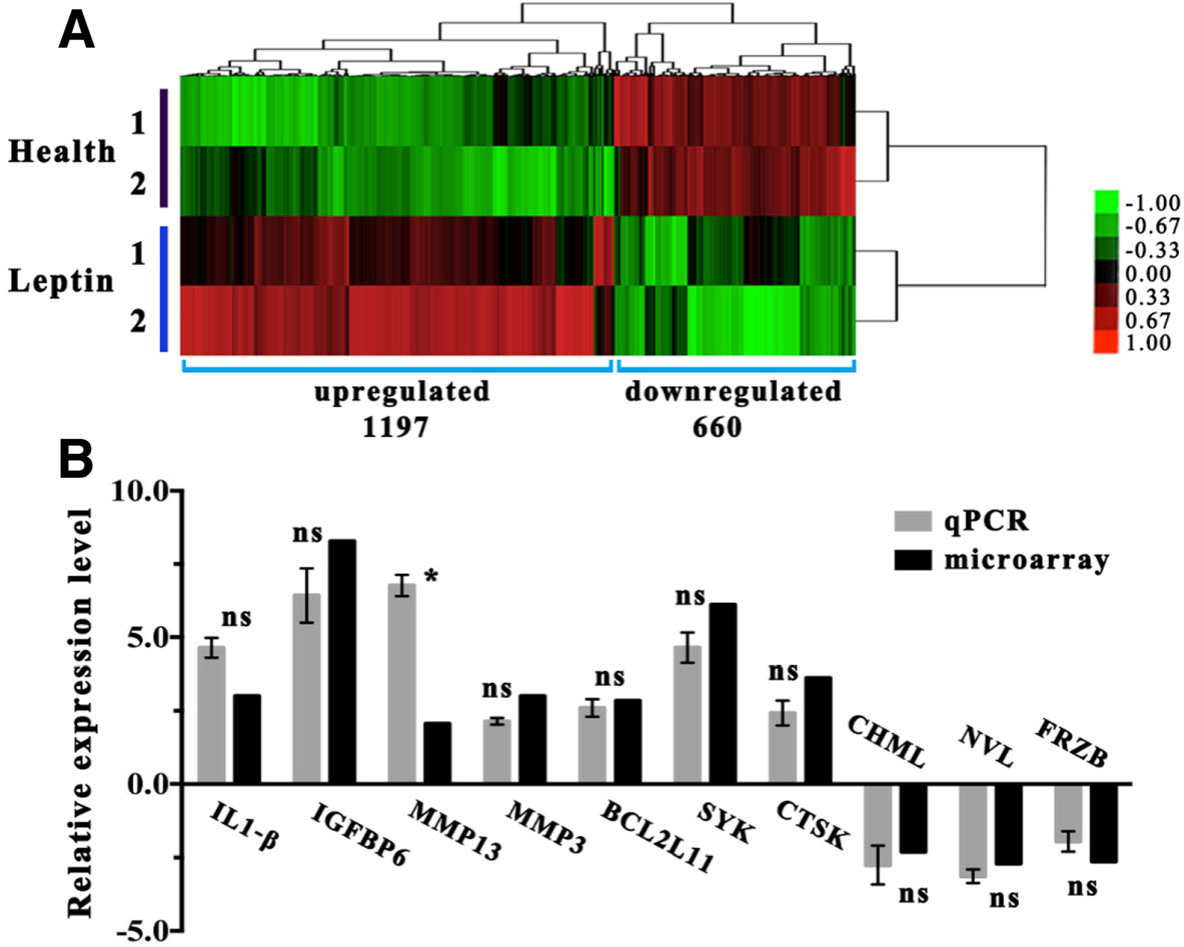
Microarray analysis and RT-qPCR verification. (**a**) Global comparison of differentially expressed genes (DEGs) in the control and leptin-induced groups is illustrated by heat maps. One-thousand and eight-hundred fifty-seven genes (*p* < 0.05, FC > 2.0) were found to be expressed at significantly different levels between the two groups, including 1197 upregulated and 660 downregulated genes. Upregulated genes are shown in the heat map in *red color*, down-regulated ones are in *green color*; evenly expressed genes are in *black color*. (**b**) Verification of microarray data on RT-qPCR. *IL-1β, IGFBP6, MMP13, MMP3, BCL2L11, SYK* and *CTSK* were up-regulated*,* while *CHML, NVL* and *FRZB* were down-regulated in the leptin-induced group when compared with the control group. Relative expression level was calculated as the mean value of six samples isolated from six different rats by using the delta Ct method. Expressions were normalized relative to that of the reference gene (β-actin). * indicates *p* < 0.05; ns = non- significant

Genome wide gene expression profiles were compared between the two groups to identify genes that showed altered expression levels in response to leptins. A total of 1857 genes (*p* < 0.05, FC > 2.0) were significantly different between the two groups, of which 1197 were up-regulated and 660 genes were down-regulated (Fig. 2a). Among the 1857 DEGs, the top 10 most significantly up-regulated genes were *ELANE, CTSG, RHAG, S100A8, SAAL1, NP4, SRGN, NKG7, PRTN3* and *S100A9*, while the top 10 most obviously down-regulated genes were *PCK2, MTNRLA, OLR322, ACTRT2, IRAK2, ATP2B2, PCDH15, OLR1230, CD80* and *ZBTB5* (Table 1). Some notable changes were observed in genes that are well known for their strong correlation with OA pathology, such as Wnt-inhibitor frizzled related protein beta [25] (FRZB, 2.76-fold down in leptin-induced group). The complete list of DEGs is provided in Additional file 1: Table S1.

**Table 1:** The ten most significantly up- and down-regulated genes in this study

Accession number	Gene symbol	Description	Fold change
*Ten most significantly up-regulated genes*			
NM_001106767	*ELANE*	*Rattus norvegicus* elastase, neutrophil expressed	68.85
NM_001106041	*CTSG*	Rattus norvegicus cathepsin G	58.80
NM_023022	*RHAG*	Rattus norvegicus Rh-associated glycoprotein	49.08
CA507495	*S100A8*	Rattus norvegicus S100 calcium binding protein A8	42.58
XM_003748860	*SAAL1*	Rattus norvegicus serum amyloid A-like 1	41.35
NM_173299	*NP4*	Rattus norvegicus defensin NP-4 precursor	35.68
NM_020074	*SRGN*	Rattus norvegicus serglycin	32.10
NM_133540	*NKG7*	Rattus norvegicus natural killer cell group 7 sequence	31.23
NM_001024264	*PRTN3*	Rattus norvegicus proteinase 3	28.58
NM_053587	*S100A9*	Rattus norvegicus S100 calcium binding protein	27.46
*Ten most significantly down-regulated genes*			
NM_001108377	*PCK2*	Rattus norvegicus phosphoenolpyruvate carboxykinase 2	−52.82
NM_053676	*MTNR1A*	Rattus norvegicus melatonin receptor 1A	−34.16
NM_001000762	*OLR322*	Rattus norvegicus olfactory receptor 322	−26.07
NM_001013937	*ACTRT2*	Rattus norvegicus actin-related protein T2	−20.12
NM_001025422	*IRAK2*	Rattus norvegicus interleukin-1 receptor-associated kinase	−16.18
NM_012508	*ATP2B2*	Rattus norvegicus ATPase, Ca^++^ transporting, plasma membrane 2	−13.46
NM_001271377	*PCDH15*	Rattus norvegicus protocadherin 15	−10.49
NM_001000595	*OLR1230*	Rattus norvegicus olfactory receptor 1230	−10.24
NM_012926	*CD80*	Rattus norvegicus Cd80 molecule	−7.81
NM_001106657	*ZBTB5*	Rattus norvegicus zinc finger and BTB domain containing 5	−7.70

To confirm the results of microarray study, expression of 10 selected DEGs (7 up-regulated and 3 down-regulated) was validated through RT-qPCR. Selection of these 10 genes was based on their importance in certain signaling pathways. Most of the tested genes showed similar results on microarray analysis and RT-qPCR (Fig. 2b). Primer sequences of the 10 genes are listed in supplementary material (Additional file 2: Table S2).

### GO and pathway analysis of DEGs in leptin-affected cartilage

**Fig. 3:**
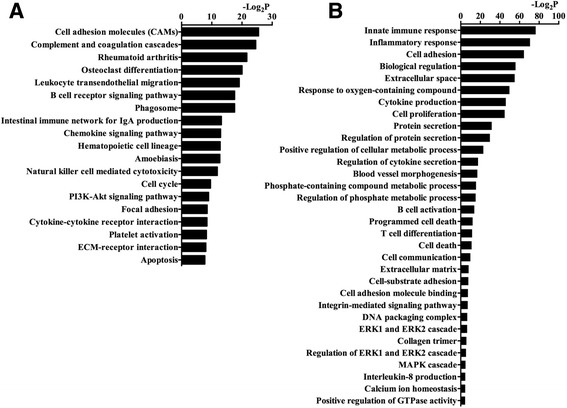
Analysis of gene ontology (GO) and cell signaling pathways. (**a**) Cell signaling pathways in rat cartilage tissue that showed significant changes after leptin induction are displayed. The vertical axis denotes the category signaling pathways, while the *horizontal axis* shows the enrichment of each pathway. (**b**) Significant GO category for regulated genes between the control and leptin-induced groups. The *vertical axis* is the GO terms, and the *horizontal axis* is the enrichment of GO. *P* < 0.05 was set as the threshold for statistical significance. -Log_2_P shows the base two logarithms of the *p* value

We defined significant KEGG pathways as those associated with *p* value <0.05. The significantly regulated pathways primarily included those involved in osteoclastic differentiation, intestinal immune network, chemokine signaling pathway, PI3K-Akt signaling pathway, cytokine-receptor interaction, ECM-receptor interaction and apoptosis (Fig. 3a). The DEGs involved in significantly regulated pathways are listed in Additional file 3: Table S3.

Significant GO categories were defined as those associated with *p* value <0.05. The GO categories of genes that were a significant between-group difference were those associated with inflammatory response, cytokine production, regulation of protein secretion, positive regulation of cellular metabolic processes, programmed cell death, extracellular matrix, ERK1/2 cascade and the MAPK cascade (Fig. 3b). One notable part of these GO terms was related to inflammation (e.g. interleukin-8 [IL-8]). Significant GO terms of DEGs and all related data are displayed in Additional file 4: Table S4.

### Comparison with previous microarray studies in OA

To further explore whether leptin is involved in the pathogenesis of OA, our leptin-mediated gene expression profile was compared with those of two previous microarray studies performed by Zhu et al. [26] and Meng et al. [27]. In the study by Zhu et al., a total of 82 differentially expressed genes were reported, many of which are known to play an important role in the pathogenesis of osteoarthritis, including inflammatory factors and matrix metalloproteinase (MMP), growth factors, apoptosis, energy and other related genes. In the second study, 138 genes or expressed sequence tags were reported as up- or down-regulated by at least 2-fold, which suggests that these genes may be involved in the progression of OA.

On data analysis and comparison with the available literature, our microarray results were divided into two groups (Table 2). On one hand, the expression of some OA-related genes including MMPs (e.g. MMP-3), inflammatory factors (e.g. IL-1β) and growth factors (e.g. IGFBP6) was significantly up-regulated by leptin induction. This suggests that leptin indeed played important roles in the pathogenesis of OA by adjusting these protein levels, which is consistent with previous reports. On the other hand, our leptin-induced gene expression profile revealed new candidate genes (e.g. *BCL2L11*) whose expression was obviously increased, but was overlooked in the previous microarray reports. Recently, *BCL2L11* (known as BIM) was shown to interact with other members of the BCL2 protein family and act as an apoptotic activator [28–30]. In the study by Zhu et al., Fas-induced chondrocyte apoptosis stimulated progression of OA, while our data indicates that leptin might mediate the onset and development of OA via activation of another pro-apoptosis gene (*BCL2L11*) rather than Fas (FC = 1.2). These findings may provide new insights into the molecular mechanisms that underlie OA and potentially unravel new therapeutic targets. Our findings suggest that leptin is an important player in the progression of OA and that its effect may be mediated via modulation of expressions of multiple related genes; however, the underlying molecular mechanisms need to be studied further.

**Table 2:** Leptin-induced genes associated with OA in this study

Accession number	Gene symbol	Description	Fold change
NM_133523	*MMP3*	Rattus norvegicus matrix metallopeptidase 3	2.87
NM_031055	*MMP9*	Rattus norvegicus matrix metallopeptidase 9	2.09
NM_133530	*MMP13*	Rattus norvegicus matrix metallopeptidase 13	2.06
NM_031512	*IL-1β*	Rattus norvegicus interleukin 1 beta	3.01
NM_012589	*IL-6*	Rattus norvegicus interleukin 6	4.66
NM_013110	*IL-7*	Rattus norvegicus interleukin 7	2.86
NM_012854	*IL-10*	Rattus norvegicus interleukin 10	3.43
NM_013104	*IGFBP6*	Rattus norvegicus insulin-like growth factor binding protein 6	8.28
NM_171988	*BCL2L11*	Rattus norvegicus BCL2-like 11 (apoptosis facilitator)	2.85
NM_031560	*CTSK*	Rattus norvegicus cathepsin K	3.61
NM_133416	*SYK*	Rattus norvegicus spleen tyrosine kinase	6.11
AF279918	*RGS2*	regulator of G-protein signaling 2	2.14
NM_001137622	*ADAMTS2*	Rattus norvegicus ADAM metallopeptidase with thrombospondin type 1 motif, 2	2.77
M12199	*COL1A1*	Rat alpha-1 type I collagen mRNA	4.01
NM_057149	*TNFSF11*	Rattus norvegicus tumor necrosis factor (ligand) superfamily, member 11	2.88
NM_001109524	*CHML*	Rattus norvegicus choroideremia-like (Rab escort protein 2)	−2.32
NM_001105980	*NVL*	Rattus norvegicus nuclear VCP-like	−2.54
NM_001100527	*FRZB*	Rattus norvegicus frizzled-related protein	−2.76

## Discussion

In the present study, microarray analyses of gene expression profiles were performed to identify candidate genes that are involved in the onset and progression of OA in a rat model. Bao et a used the same rat model of leptin-induced OA to study the expression profiles of healthy and leptin-induced cartilage [13]. However, they just used qPCR and Western Blot to study a limited number of genes. To the best of our knowledge, our study represents the first comprehensive comparison of whole genome expression profile of healthy and leptin-induced cartilage. The microarray results were validated by RT-qPCR for a selected group of genes. Several new candidate genes were found to be associated with the pathogenesis of OA for the first time.

Several recent studies have shown abnormal expressions of IL-1, TNF-α and MMPs during the pathogenesis of OA [31–34]. On analyses of GO and KEGG pathways, we confirmed the important roles of these genes in the pathogenesis of OA. IL-1 is one of the most important factors implicated in cartilage degeneration during progression of OA, and a main initiator of the pathophysiological imbalance of matrix homeostasis. IL-1 was also shown to suppress anabolism of the cartilage extracellular matrix, leading to breakdown of hyaline cartilage tissue [35]. In this study, we found that genes up-regulated by leptin were associated with the cellular response to IL-1β and IL-6. This suggested that molecular mechanism of leptin-induced OA may involve modulation of inflammatory factors. Secondly, the expression of genes encoding for catabolic enzymes (such as *MMP-3*, −*9* and −*13*) was significantly up-regulated by leptin induction, which further supports the involvement of leptin in the pathogenesis of OA. Various experimental studies have documented high expression levels of MMPs in the OA cartilage. Further, endogenous MMP inhibitors have been shown to delay the progression of OA [36].

Growth factors are a group of proteins that affect the cell behavior through cellular signal transduction. In articular joints, chondrocytes produce copious amounts of growth factors which are stored in the extracellular matrix. These growth factors can in turn regulate the proliferation, differentiation and metabolism of chondrocytes [37]. Changes in the expressions of some of these growth factors, such as Insulin-like growth factor binding protein 6 (IGFBP6, FC = 8.28), upon stimulation by leptin is well documented. In a recent in vitro study, IGFBP-6 was shown to inhibit the actions of IGF-II as indicated by attenuation of IGF- II-induced proliferation, differentiation, migration and survival [38]. Pleiotropic effects of insulin-like growth factors (IGFs) on many types of cells are well documented; for example, these were shown to inhibit apoptosis through the PI3K/Akt pathway, and to promote mitogenesis via the Ras/MAPK pathway [39]. Therefore, up-regulation of IGFBP6 in leptin-induced cartilage may induce catabolic activity in the cartilage matrix via inhibition of the protective effects of IGF-II. In addition, member 11 of the tumor necrosis factor ligand superfamily member (TNFSF 11), which is a driver of osteoclast differentiation and bone resorption, appeared to be up-regulated in leptin-induced cartilage. This observation is consistent with the results of a previous OA-related study [40].

Interestingly, available evidence suggests that two different pathways are involved in the apoptosis of chondrocytes during the progression of OA [41, 42]. One of these pathways is nitric oxide (NO) independent. The other one is associated with synovitis and is mediated by *Fas*. However, our results indicated that leptin might activate the apoptosis of chondrocytes and lead to the onset and development of OA via a new candidate pro-apoptotic gene (*BCL2L11*, FC = 2.9) rather than *Fas* (FC = 1.2).

## Conclusion

To summarize, we analyzed the microarray data to compare the gene expression profiles of healthy cartilage and leptin-induced cartilage, and identified a number of DEGs and expression patterns. Moreover, our findings provide some novel insights into the crucial role of leptin in the molecular mechanisms that underlie OA pathogenesis, even though it needs further evaluation.

## Additional files

Additional file 1: Table S1. Complete list of DEGs between control and leptin-induced groups. (XLSX 568 kb)

Additional file 2: Table S2. Sequences of primer used. (DOC 43 kb)

Additional file 3: Table S3. Significant signaling pathways analysis of the DEGs. (XLSX 14 kb)

Additional file 4: Table S4. A full list of significant GO terms of DEGs. (XLSX 129 kb)
