# Supplementary material for: Microarray study of gene expression profile to identify new candidate genes involved in the molecular mechanism of leptin-induced knee joint osteoarthritis in rat
Source: Hereditas. 2017 Jul 4;155:4. doi: 10.1186/s41065-017-0039-z (PMC5496599; doi:10.1186/s41065-017-0039-z)
Supplement: Supplementary file 2 — Sequences of primer used. (DOC 43 kb) [file 41065_2017_39_MOESM2_ESM.doc]

**Table S2. Primers used in the present** study

| **Primer^a^** | **Nucleotide sequence** |
| --- | --- |
| IL1-βF | 5´-acctctcaagcagagcacaga-3´ |
| IL1-βR | 5´-AGCCATCTTTAGGAAGACACG-3´ |
| IGFBP6F | 5´-acgaagagacacctttgcgg-3´ |
| IGFBP6R | 5´-TGCTCTCCTTGGTCGTCTCT-3´ |
| MMP13F | 5´-gacacagagatggagaaattc-3´ |
| MMP13R | 5´-CTATGCTATTGCTTAGGATCC-3´ |
| MMP3F | 5´-agcatgcagtatctacatgact-3´ |
| MMP3R | 5´-AGAATCAGGTGATCCTTGGG-3´ |
| Bcl2l11F | 5´-gagagtgtatctttgacaactc-3´ |
| Bcl2l11R | 5´-CCCCACCTGCCCTTTTCTT-3´ |
| SykF | 5´-aaggattgtgactatatttgcc-3´ |
| SykR | 5´-CTGGAAACTTGCTTGTAAGTAT-3´ |
| CtskF | 5´-aggcagctaagtgcagagg-3´ |
| CtskR | 5´-TACGATGGACACAGAGACGG-3´ |
| ChmlF | 5´-actggagaaagaaaagttctgt-3´ |
| ChmlR | 5´-GCAAGTTTGTGTTCACCTTCT-3´ |
| NvlF | 5´-gatttggaagcaattgctaatg-3´ |
| NvlR | 5´-TCAGGGCACAGAGAGAAGC-3´ |
| FrzbF | 5´-accgcaggaggagaatcca-3´ |
| FrzbR | 5´-ACCATGGTCCAGCAGGAT-3´ |

^a^ All primers were obtained from Invitrogen (Shanghai, China).
